# 重型/极重型再生障碍性贫血患者免疫抑制治疗后早期死亡的临床特征及预测模型构建

**DOI:** 10.3760/cma.j.issn.0253-2727.2022.11.006

**Published:** 2022-11

**Authors:** 苗 陈, 俊玲 庄, 辰 杨, 为 王, 炎 张, 路 张, 丹青 赵, 俊 冯, 剑 李, 道斌 周, 冰 韩

**Affiliations:** 中国医学科学院、北京协和医学院北京协和医院血液科，北京 100730 Department of Hematology, Peking Union Medical College Hospital, Peking Union Medical College & Chinese Academy of Medical Sciences, Beijing 100730, China

**Keywords:** 贫血，再生障碍性, 抗胸腺细胞球蛋白, 早期死亡, Anemia, aplastic, Antithymocyte immunoglobulin, Early death

## Abstract

**目的:**

分析重型/极重型再生障碍性贫血（SAA/VSAA）采用抗胸腺细胞球蛋白（ATG）强化免疫抑制治疗后早期死亡（ED）患者的特征并构建ED的预测模型。

**方法:**

收集2003年8月至2021年8月期间在北京协和医院接受ATG治疗的232例SAA/VSAA患者临床资料，回顾性分析ED（治疗后90 d内死亡）患者的临床特征、死亡原因，采用Cox比例风险模型筛选影响ED的危险因素并构建预测模型。

**结果:**

232例SAA/VSAA患者接受ATG治疗，19例（8.2％）发生ED，中位发生时间为24（3～85）d。ED主要原因是感染（84.2％），其次为脑出血（10.5％）。多因素分析显示VSAA（*HR*＝15.359，95％ *CI* 1.935～121.899，*P*＝0.010）、采用泊沙康唑预防真菌感染（*HR*＝0.147，95％ *CI* 0.019～1.133，*P*＝0.066）、外周血淋巴细胞计数（LYM）≤0.5×10^9^/L（*HR*＝3.386，95％*CI* 1.123～10.206，*P*＝0.030）、PLT≤5×10^9^/L（*HR*＝8.939，95％ *CI* 1.948～41.019，*P*＝0.005）为ED的独立影响因素。VSAA、采用泊沙康唑预防真菌感染、LYM≤0.5×10^9^/L、PLT≤5×10^9^/L分别赋3、−2、1、2分构建临床预测模型，该积分模型曲线下面积（AUC）为＝89.324（95％*CI* 80.859～97.789），积分≥3分患者发生ED的风险为<3分组的23.1（95％*CI* 5.3～100.2）倍。

**结论:**

感染和脑出血导致的ED是SAA/VSAA采用ATG治疗的重要挑战。同时具有VSAA、LYM≤0.5×10^9^/L、PLT≤5×10^9^/L且未采用泊沙康唑预防真菌感染患者具有较高的ED发生风险。

## Abstract (en)

**Objective:**

Early death（ED）characteristics and predictive factors analysis in patients with severe/very severe aplastic anemia（SAA/VSAA）treated with intensive immunosuppression therapy and establish an ED prediction model.

**Methods:**

The clinical data of 232 patients with SAA/VSAA treated with Antithymocyte immunoglobulin（ATG）at the Peking Union Medical College Hospital from August 2003 to August 2021 were collected. The characteristics and causes of ED within 90 days were analyzed retrospectively. Cox proportional hazards model was used to screen the ED risk factors and build a prediction model.

**Results:**

Only 19 patients（8.2％）developed ED with a median time of 24（3–85）days among the 232 patients with SAA/VSAA who received ATG treatment. The main cause of ED was infection（84.2％）, followed by cerebral hemorrhage（10.5％）. Multivariate analysis showed that VSAA（*HR*＝15.359, 95％ *CI* 1.935–121.899, *P*＝0.010）, fungal infection prevention by posaconazole（*HR*＝0.147, 95％ *CI* 0.019–1.133, *P*＝0.066）, lymphocyte count（LYM）≤ 0.5×10^9^/L（*HR*＝3.386, 95％ *CI* 1.123–10.206, *P*＝0.030）, and PLT ≤ 5×10^9^/L（*HR*＝8.939, 95％ *CI* 1.948–41.019, *P*＝0.005）were ED's independent influencing factors. To build a clinical prediction model, VSAA, fungal infection prevention by posaconazole, LYM ≤ 0.5×10^9^/L, and PLT ≤ 5×10^9^/L were scored with 3, −2, 1, and 2, respectively. The integral model AUC＝89.324（95％ *CI* 80.859–97.789）. The ED risk in patients with a score ≥ 3 was 23.1（95％ *CI* 5.3–100.2）times that in patients with a score<3.

**Conclusion:**

ED caused by infection and cerebral hemorrhage is an important challenge for SAA/VSAA to be treated with ATG. VSAA, LYM ≤ 0.5×10^9^/L, and PLT ≤ 5×10^9^/L patients who did not use posaconazole to prevent fungal infection had a high ED risk.

对于年龄大于40岁或无同胞全相合供者的重型/极重型再生障碍性贫血（SAA/VSAA）患者，一线治疗为抗胸腺细胞球蛋白（ATG）联合环孢素A（CsA）的强化免疫抑制治疗（IST）[1]–[2]。但IST起效时间需1～3个月，由于患者存在严重全血细胞减少，ATG治疗后免疫功能进一步下降，存在早期死亡（ED）风险。本研究我们回顾性分析采用ATG治疗的SAA/VSAA患者发生ED的临床特征及其影响因素，并通过危险因素构建ED的临床预测模型，为尽早识别高危患者提供参考。

## 病例与方法

1. 病例资料：纳入2003年8月至2021年8月期间在北京协和医院接受ATG治疗的SAA/VSAA患者。患者均无血液病家族史或躯体畸形，部分患者行染色体断裂试验和彗星试验排除先天性AA。AA诊断及分型参照修订的Camitta标准[3]–[4]，SAA符合以下3条标准中至少2条：ANC<0.5×10^9^/L，PLT<20×10^9^/L，网织红细胞计数（Ret）<20×10^9^/L。ANC<0.2×10^9^/L定义为VSAA。急性SAA定义为SAA Ⅰ型；慢性AA病程中病情恶化，临床、血常规、骨髓象与急性SAA相同，定义为SAA Ⅱ型[5]。合并阵发性睡眠性血红蛋白尿症（PNH）克隆定义为流式细胞术检测粒细胞CD59阴性克隆≥1％。启动ATG治疗时有合并症定义为ATG治疗前一周内有合并症，包括感染、脏器出血、肝功能异常（ALT>2×ULN）、肾功能异常（血肌酐>1×ULN）。ULN代表正常值上限。

所有存活患者ATG治疗后随访时间大于3个月。收集患者临床数据，包括疾病诊断、治疗前后实验室检查资料、治疗反应、生存情况。

2. 治疗方法：ATG为基础的强化IST方案：①猪抗人淋巴细胞免疫球蛋白（pALG）20～30 mg·kg^−1^·d^−1^，共5 d；或兔抗人胸腺细胞免疫球蛋白（rATG）3～5 mg·kg^−1^·d^−1^，共5 d。予甲泼尼龙预防血清病。②CsA：起始剂量3～5 mg·kg^−1^·d^−1^，分两次口服，维持CsA血浆谷浓度200～400 µg/L。③部分患者联合应用小分子促血小板生成素受体激动剂（TPO-RA）艾曲泊帕或海曲泊帕、雄激素促进造血。④支持治疗：抗感染治疗，输注红细胞悬液、血小板，G-CSF输注。给予口服氟康唑、伊曲康唑或泊沙康唑预防真菌感染，口服复方磺胺甲恶唑预防肺孢子虫感染。

3. 血液学疗效判定：部分缓解（PR）：摆脱输血依赖，不符合SAA标准；完全缓解（CR）：血常规基本恢复正常，即ANC>1×10^9^/L，HGB>110 g/L（女性）或>120 g/L（男性），PLT>100×10^9^/L。总反应包括PR及CR[5]。

4. 研究终点的定义：主要研究终点为ED，定义为ATG治疗后90 d内死亡。次要终点为血液学疗效及总生存（OS），OS定义为ATG治疗第1天到死亡（事件）或末次随访（删失）的时间。随访截至2021年11月1日。

5. 统计学处理：采用SPSS 26.0进行统计分析。分类变量组间比较采用Fisher精确概率法，连续变量组间比较采用非参数检验。通过受试者工作特征分析（ROC曲线）约登指数确定连续变量cut-off值作为分界值。采用Kaplan-Meier法绘制生存曲线，采用Cox比例风险模型对以下参数进行预后影响因素评估：性别、年龄（<40岁和≥40岁）、AA分型（SAA和VSAA）、病因（肝炎相关和特发性）、治疗时间（2017年之前和2017年之后）、启动ATG治疗前一周内有无合并症、启动ATG治疗时有无粒细胞缺乏（粒缺）、ATG类型（pATG和rATG）、是否联合TPO-RA、是否采用真菌预防用药、是否采用泊沙康唑预防真菌感染、是否存在PNH克隆、淋巴细胞计数（LYM）、Ret、PLT。单因素分析中*P*<0.10的预后因素纳入多因素分析，多因素通过逐步法进行变量筛选，根据赤池信息准则（AIC）最小化原则构建预测模型，采用曲线下面积（AUC）评价模型区分度。双侧*P*<0.05为差异有统计学意义。

## 结果

1. 一般临床特征：共纳入232例SAA/VSAA患者，中位年龄34（12～76）岁，其中<40岁135例（58.2％）、≥40岁97例（41.8％）。男性133例（57.3％），女性99例（42.7％）。SAA 147例（63.4％），VSAA 85例（36.6％）；特发性AA 216例（93.1％），肝炎相关AA 16例（6.9％）；SAA-Ⅰ型221例（95.3％），SAA-Ⅱ型11例（4.7％）。62例（26.7％）伴PNH克隆。中位HGB 61（26～138）g/L，中位WBC 1.57（0.12～5.70）×10^9^/L，中位ANC 0.31（0.00～3.17）×10^9^/L，中位LYM 1.15（0.00～4.24）×10^9^/L，中位PLT 9（1～43）×10^9^/L，中位Ret 13.2（1.1～125.8）×10^9^/L。采用pATG治疗161例（69.4％），中位剂量25.00（11.54～30.49）mg·kg^−1^·d^−1^；rATG治疗71例（30.6％），中位剂量3.14（2.22～3.75）mg·kg^−1^·d^−1^。9例启动ATG治疗时已合并真菌感染，采用抗真菌治疗，未纳入真菌预防相关分析。

2. ED发生情况：232例SAA/VSAA患者中19例（8.2％）发生ED，中位发生时间24（3～85）d，其中11例在30 d内死亡。

19例发生ED患者的中位年龄35（19～63）岁，男性9例（47.4％），女性10例（52.6％）。起病到ATG治疗中位时间1（0.3～108）个月。SAA 2例（10.5％），VSAA 17例（89.5％）。肝炎相关AA 2例（10.5％），特发性AA 17例（89.5％）。SAA Ⅰ型18例（94.7％），SAA Ⅱ型1例（5.3％）。rATG治疗组4例（21.1％），pATG治疗组15例（78.9％）。伴PNH克隆4例（21.1％），不伴PNH克隆15例（78.9％）。3例启动ATG治疗时已经合并真菌感染，余16例患者中11例接受真菌预防药物（泊沙康唑1例、伊曲康唑5例、氟康唑5例），5例未接受真菌预防药物。16例（84.2％）在启动ATG治疗前一周内有合并症：9例（肺部感染2例、鼻窦感染1例、发热无明确感染灶6例）抗感染治疗有效，启动ATG治疗时体温已控制；1例大脑、小脑多发硬膜下出血。6例（肺部感染2例、发热无明确感染灶3例、皮肤红肿脓疱1例）感染未控制。1例患者肝功能异常。

16例（84.2％）患者死于感染，其中肺部细菌感染2例、肺部真菌感染3例、肺部混合感染1例、血流感染3例、血流合并肺部真菌或混合感染4例（其中1例左房菌栓脱落猝死）、急性胆囊炎合并血流感染1例、发热感染灶不明2例。2例（10.5％）死于脑出血，其中1例脑出血伴消化道出血、肺部感染、血流感染。1例（5.3％）死亡原因不明。19例ED患者血流感染病原学培养阳性包括全耐药鲍曼不动杆菌、肺炎克雷伯菌、缓症链球菌、屎肠球菌、泛耐药鲍曼不动杆菌、克柔念珠菌、鲍曼不动杆菌、铜绿假单胞菌，肺部病原学涂片或培养阳性包括丝状真菌、毛霉菌、产酸克雷伯菌。

3. ED影响因素分析：30、60、90 d全部患者的ED率分别为（4.7±1.4）％、（6.5±1.6）％和（8.2±1.8）％。单因素分析结果显示VSAA（*P*<0.001）、2017年之后接受治疗（*P*＝0.031）、启动ATG治疗前一周内有合并症（*P*＝0.002）、启动ATG时伴粒缺（*P*＝0.021）、LYM≤0.5×10^9^/L（*P*<0.001）、PLT≤5×10^9^/L（*P*＝0.001）、Ret≤5×10^9^/L（*P*＝0.001）是发生ED的影响因素。本研究2017年开始IST联合TPO-RA，共37例患者接受TPO-RA治疗，其中艾曲泊帕24例、海曲泊帕13例，单因素结果显示是否联合TPO-RA并非ED的影响因素，治疗时间为2017年之前是ED的危险因素，但因治疗时代不能成为ATG治疗前的风险评估指标，故未纳入多因素分析。将其他单因素分析*P*<0.1的因素纳入多因素分析，采用逐步法进行变量筛选，结果显示VSAA（*β*＝2.732，*HR*＝15.359，95％ *CI* 1.935～121.899，*P*＝0.010）、采用泊沙康唑预防真菌感染（*β*＝−1.918，*HR*＝0.147，95％ *CI* 0.019～1.133，*P*＝0.066）、LYM≤0.5×10^9^/L（*β*＝1.220，*HR*＝3.386，95％*CI* 1.123～10.206，*P*＝0.030）、PLT≤5×10^9^/L（*β*＝2.190，*HR*＝8.939，95％ *CI* 1.948～41.019，*P*＝0.005）进入最终模型，为影响ED的独立影响因素（表1）。

**表1:** 232例接受ATG治疗的SAA/VSAA患者早期死亡的预后影响因素

因素	单因素分析		多因素分析	
	*HR*（95% *CI*）	*P*值	*HR*（95% *CI*）	*P*值
年龄≥40岁	0.805（0.317~2.045）	0.648		
男性	0.650（0.264~1.599）	0.348		
VSAA	16.438（3.797~71.171）	<0.001	15.359（1.935~121.899）	0.010
特发性AA	0.605（0.140~2.617）	0.501		
治疗时间为2017年之后	0.110（0.015~0.822）	0.031	-	-
ATG治疗前一周有合并症	7.036（2.050~24.150）	0.002		
ATG治疗时伴粒细胞缺乏	10.687（1.427~80.060）	0.021		
pATG	1.710（0.567~5.151）	0.341		
联合TPO-RA	0.240（0.032~1.786）	0.163		
给予真菌预防用药	1.310（0.455~3.771）	0.616		
采用泊沙康唑预防真菌感染	0.157（0.021~1.188）	0.073	0.147（0.019~1.133）	0.066
伴PNH克隆	0.725（0.240~2.183）	0.567		
LYM≤0.5×10^9^/L	5.798（2.354~14.278）	<0.001	3.386（1.123~10.206）	0.030
PLT≤5×10^9^/L	4.565（1.797~11.597）	0.001	8.939（1.948~41.019）	0.005
Ret≤5×10^9^/L	5.078（1.890~13.640）	0.001		

注 ATG：抗胸腺细胞球蛋白；SAA：重型再生障碍性贫血；VSAA：极重型再生障碍性贫血；pATG：猪抗人淋巴细胞免疫球蛋白；TPO-RA：血小板生成素受体激动剂；PNH：阵发性睡眠性血红蛋白尿；LYM：淋巴细胞计数；Ret：网织红细胞计数；-：不适合

4. 预测模型构建：由于多因素分析结果示采用泊沙康唑预防真菌感染的*P*值为0.066>0.05，纳入VSAA、采用泊沙康唑预防真菌感染、LYM≤0.5×10^9^/L、PLT≤5×10^9^/L构建为模型1，纳入VSAA、LYM≤0.5×10^9^/L、PLT≤5×10^9^/L构建为模型2，因模型1的AIC小于模型2（102对106），确定模型1更优。根据回归系数进行评分，VSAA、采用泊沙康唑预防真菌感染、LYM≤0.5×10^9^/L、PLT≤5×10^9^/L分别为3、−2、1、2分，积分范围为−2～6分。该积分模型AUC＝89.324（95％*CI* 80.859～97.789），具有较好区分度。以3分为界值将患者分为两组，积分<3分与积分≥3分组ED率分别为（1.2±0.8）％、（24.6±5.2）％（*P*<0.001），积分≥3分患者发生ED的风险为<3分组的23.1（95％*CI* 5.3～100.2）倍（图1）。

**图1:**
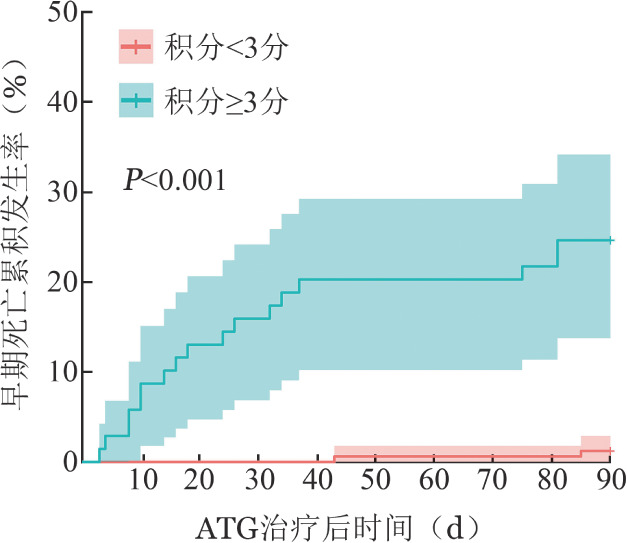
ATG治疗后早期死亡积分<3分与≥3分重型/极重型再生障碍性贫血患者生存曲线 注 ATG：抗胸腺细胞球蛋白

5. 疗效及转归：本组SAA/VSAA患者采用ATG治疗，3个月总反应率（ORR）为60.3％，CR率为4.3％；6个月ORR为73.3％，CR率为22.4％。中位随访59.3（0.1～204.5）个月，至6个月时共20例（8.6％）死亡，即3～6个月间仅增加1例死亡病例，该患者疗效为NR。预计5年OS率为88.1％，预计10年OS率为83.0％。截止随访终点，10例（4.3％）患者进展为骨髓增生异常综合征/急性髓系白血病（MDS/AML），14例（6.0％）患者进展为PNH。

## 讨论

自1888年Paul Ehrlich首次描述年轻孕妇严重贫血、出血和高热最终死亡，1904年Chauffard引入术语AA，直到20世纪60年代晚期异基因造血干细胞移植进入到AA治疗之前，SAA因骨髓造血衰竭、严重全血细胞减少，一直具有致死性特征[6]。移植技术的发展显著改善了SAA患者预后，20世纪70年代出现ATG，随着抗感染治疗、输血等支持治疗的进步，目前ATG联合环孢素A、艾曲泊帕治疗初治SAA的6个月总反应率可以达到92％[7]。SAA患者的疗效和生存已经显著改善。

但ATG治疗起效一般需要1～3个月时间，在此期间，严重感染和出血可能造成患者ED，失去等待后续ATG起效的时机以及即使ATG无效行移植挽救治疗的机会。了解ED患者特征并早期识别，采用有效措施降低ED能进一步提高ATG疗效。

一项欧洲和亚洲多中心回顾性研究中[8]，纳入955例2001–2012年间一线采用rATG治疗的AA患者，90 d内死亡率为4％，死亡原因为病原不明感染、出血和真菌感染。ED与年龄及疾病严重程度相关，60岁以上患者ED率高达16.3％，VSAA患者ED率为7％，显著高于SAA/NSAA（3％）。近年ED率显著下降（2001–2008年与2009–2012年相比，ED率分别为4％和2％，*P*<0.001）。另一项巴西的多中心回顾性研究[9]纳入2000–2014年间采用rATG治疗的185例SAA患者，ED率为15.1％。多因素分析显示年龄>35岁（*HR*＝5.06，95％ *CI* 1.87～13.69，*P*＝0.001）、基线ANC≤0.1×10^9^/L（*HR*＝7.64，95％ *CI* 3.05～19.15，*P *<0.001）为rATG治疗后ED的危险因素。

本研究中232例SAA/VSAA患者ATG治疗后90 d ED率为（8.2±1.8）％，共19例患者死亡，3个月后到6个月间仅增加1例ATG无效死亡病例，说明3个月内是ATG治疗高风险期。VSAA患者ED风险是SAA患者的15.359倍。VSAA患者骨髓衰竭更严重，往往血象更低，更多患者启动ATG治疗时已存在合并症、对G-CSF无反应持续粒缺。使用G-CSF后中性粒细胞上升往往提示骨髓有残存造血功能，而且摆脱粒缺后进入ATG治疗感染风险明显下降，本组患者中启动ATG治疗时不粒缺的83例患者无一例发生ED。可见分型为SAA、使用G-CSF后不粒缺的患者采用ATG治疗安全性高。以往的研究中[8]–[9]年龄往往是ED的高危因素，本研究中<40岁和≥40岁患者ED率差异无统计学意义，19例发生ED的患者中位年龄仅为35（19～63）岁，提示本组患者中年龄并非ED的影响因素。

本研究中，2017年之后治疗的患者ED率显著下降。原因之一是2015开始采用泊沙康唑预防真菌感染，之前为采用氟康唑、伊曲康唑预防。泊沙康唑抗菌谱广且强效，且对SAA患者ATG治疗过程中多种合并用药的作用比伊曲康唑小[10]–[11]。在整组患者中采用泊沙康唑预防真菌已显示出降低ED的趋势，纳入模型具有更好的预测效能。其次，2017年开始联合使用TPO-RA，本研究中共37例联合艾曲泊帕或海曲泊帕患者无一例发生ED。有研究显示IST联合TPO-RA可加快起效时间、提高ORR及CR率[12]。但本研究尚无法得出TPO-RA是ED的保护因素（*P*＝0.163）。

目前SAA患者首选移植或者ATG治疗的依据仍只是年龄和有无同胞供者[1]–[2]，需要整合更多特征更精细的分层来个体化选择治疗方案。如能识别出ATG治疗早期死亡的高危患者，早期行移植治疗预计能改善这部分患者预后。本研究纳入VSAA、泊沙康唑预防真菌感染、LYM≤0.5×10^9^/L、PLT≤5×10^9^/L构建了预测ED积分系统，积分≥3分的患者ED率高达24.6％，提示采用ATG治疗预后差，应考虑其他治疗方案从而减少早期死亡的发生。
